# Integrative Pediatrics: Successful Implementation of Integrative Medicine in a German Hospital Setting—Concept and Realization

**DOI:** 10.3390/children5090122

**Published:** 2018-09-04

**Authors:** Marion Eckert, Catharina Amarell, Dennis Anheyer, Holger Cramer, Gustav Dobos

**Affiliations:** 1 Kinderkrankenhaus St. Marien, Grillparzerstr. 9, 84036 Landshut, Germany; catharina.amarell@st-marien-la.de; 2 Department of Internal and Integrative Medicine, Kliniken Essen-Mitte, Faculty of Medicine, University of Duisburg-Essen, Am Deimelsberg 34a, 45276 Essen, Germany; D.Anheyer@kliniken-essen-mitte.de (D.A.); H.Cramer@kliniken-essen-mitte.de (H.C.); g.dobos@kliniken-essen-mitte.de (G.D.)

**Keywords:** integrative medicine, pediatrics, clinical practice

## Abstract

Complementary and Alternative Medicine (CAM) has not been systematically institutionalized in pediatric hospital care in Germany so far. For the responsible implementation and systematic evaluation of CAM in pediatric care, a model project was initialized in three different pediatric hospitals in Germany, one of them being the “Kinderkrankenhaus St. Marien” in Landshut, Germany. During this project, a concept of the implementation process was developed based on clinical care, teaching, and scientific evaluation. A project group was formed in St. Marien, which included leaders of the hospital, physicians, nurses, and physiotherapists. Over a period of three years, pediatric treatment modalities of the CAM-spectrum were systematically integrated into routine pediatric care and a new integrative medicine department was established. CAM is now being applied in an inpatient as well as outpatient setting, in addition to conventional medical treatments. The modalities now applied include Traditional Chinese Medicine (TCM), relaxation, hypnosis, reflexology, wraps and poultices, aromatherapy, homeopathy, yoga, and herbal medicine. Studies were initiated in some areas. The process and concept leading up to this successful implementation will be described in this article. We show that with motivated team players and structured proceedings, implementation of integrative medicine in a children’s hospital can be successful.

## 1. Introduction

Integrative Medicine (IM) is an emerging field, even if the increasingly wide use of Complementary and Alternative Medicine (CAM) and its incorporation into conventional medicine has been described in numerous publications [1,2,3]. IM incorporates the use of methods of the CAM spectrum into conventional medicine in an evidence-based way and integrates the whole person and the environment that person lives in into the treatment of adults as well as children. The Academic Consortium of Integrative Medicine & Health (ACIMH) states that “Integrative Medicine is the practice of medicine that reaffirms the importance of the relationship between practitioner and patient, focuses on the whole person, is informed by evidence, and makes use of all appropriate therapeutic approaches, healthcare professionals and disciplines to achieve optimal health and healing” [4].

Many authors have described the substantial increase in the use of CAM, not only in adults, but also in children [3,5,6,7,8,9,10,11], as well as the lack of information the treating physicians receive about the methods used and the possible side-effects that some of these treatments may have [12,13,14]. A variety of treatment modalities are nowadays applied in integrative medicine in the field of pediatrics [2]. Teaching programs for physicians and residents have been developed to increase their knowledge about and widen their spectrum of CAM treatments [15,16,17]. Many institutions have implemented integrative medicine in various models, even in hospitals [1,18,19,20]. Some of them have identified important factors and strategies that are vital for successful implementation [18,19]. Yet, CAM has not been widely systematically institutionalized in pediatric care in Germany so far—in hospitals or private practice.

Our intent was to implement integrative medicine in a pediatric hospital, by building a concept upon our and other’s experiences that can be applicable and useful for different hospitals that strive to develop such programs. We also aimed at contributing to scientific insights on the modalities used. Therefore, at the very start of the project, an overview regarding the scientific situation of CAM in pediatrics was compiled and possible indications for CAM in children identified in a multidisciplinary meeting.

## 2. The Hospital Setting of St. Marien

The Children’s Hospital “Kinderkrankenhaus St. Marien” in Landshut, Germany, is an academic teaching hospital of the LMU (Ludwig-Maximilians-University) München. It is equipped with 120 beds. An average of 6500 children are treated annually on the wards and about 16,000 children in the specialized outpatient departments. Seventeen thousand children are seen in the pediatric emergency department annually. A social pediatric center, which takes care of about 5200 patients per year, is affiliated with the hospital.

St. Marien Hospital has always been dedicated to promoting integrative approaches for the medical treatment of children. Besides conventional medicine, additional certifications of attending physicians allowed for naturopathic and homeopathic treatments, even in the years before our project started. For the past 10 years for example, a pediatrician with certification in homeopathy has been employed in the hospital treating inpatients as well as children in the outpatient department. A pediatrician certified in naturopathic medicine had also established policies for the application of naturopathic treatments for pertinent indications like upper airway infections or certain pain conditions.

## 3. Preparation

Whenever an institution takes a new direction, it is important that its leaders make decisions on the new course of action to be taken from then on. This needs to be communicated and future expectations made clear to the rest of the organization. This also applies to establishing an integrative medical care model within a hospital. Only then does the body of the organization know that a new corporate strategy is being pursued and employees are expected to act upon it in certain ways. In addition, a decision needs to be made on the areas in which CAM is supposed to be integrated in the hospital. The areas can be clinical, teaching, or research [19], depending on the structure of the hospital and interests of the organization. Not every institution will want to or be able to serve all three areas and this is not necessary. Various models, from e.g., focusing on pain management, research orientation, or offering a wide variety of integrative care modalities to patients, can work [19]. Furthermore, there are different approaches that can be taken in order to execute the new strategy. One is a model from “inside” the institution; the other would be a more external approach in which new staff with new skills is employed to start a new venture. We chose the model from inside, in which we built upon the skills and motivation of staff members. This offers the advantage of using existing resources and preventing resistance from staff members on new treatment methods. This has been described as one of the cornerstones of a successful implementation [19].

## 4. First Steps

First, a project group with members of different disciplines of the hospital with one coordinating leader was formed. This group met once a month to determine next steps to be taken and goals to accomplish. It was beneficial to have the executive director of the hospital as a part of the group, amongst attending physicians of different disciplines, nursing management, nurses, physiotherapists, and psychologists in order to reach staff members from different disciplines and to build integrative medicine from inside the organization. A new sense of a common comprehensive project led to the necessary enthusiasm in all participants. It was important for the coordinating leader to be an experienced senior pediatrician, also trained in a broad field of complementary medicine, and someone who was accepted by all members of the project group. This leader was responsible for coordinating activities, establishment of contacts, development of next steps, creating questionnaires, communication with other facilities and researchers, networking, and motivation. Leaders from other pediatric integrative medicine programs were contacted and literature was reviewed for advice and ideas on how to successfully establish integrative medicine within a hospital [18,19,21].

The second step involved the determination of resources existing within the hospital. Hospital staff—be it nurses, doctors or physiotherapists—have a variety of additional qualifications in the area of complementary medicine, which they would readily apply in their work. To determine the existing resources, a questionnaire was designed, as other centers had done before in a similar way [22]. The questionnaire included questions about additional qualifications, overall interest in CAM being introduced in the hospital, and interest to use those qualifications in the treatment of children. We found that there was an immense variety of knowledge and skills already present in St. Marien (Table 1), similar to modalities applied in other centers in the US [2]. It was these resources that we utilized to build upon in the process of implementing IM in our institution.

Thirdly we were interested in the assessment of desires of parents. Many studies showed that CAM methods are readily applied in children and that parents also desire the use of CAM in the treatment of their children [3,23]. There are regional differences though, so we wanted to know about the wishes of the parents in the local area as well as their past experiences with CAM and willingness to make additional payments for the integration of CAM in the treatment of their child. Therefore, another questionnaire was created and given to the parents arriving at the hospital. The majority of parents were very approving of CAM treatments for their children. These data will be published in a separate article and therefore will not be further described.

The fourth step was to start educating the nurses, physicians and other staff on different kinds of CAM methods. It is an advantage if there is a culture of regular teaching schedules (like lunch symposia or in-house trainings) already established within the institution. In St. Marien, CAM trainings were included in the in-house trainings taking place regularly. Teachings about aromatherapy, foot reflexology, wraps and poultices, homeopathy, hypnosis, and naturopathic treatment options were given by experts (physicians and nurses) this way. Instructions on the application of acupuncture needles, acupressure, aromatherapy, and homeopathy were also given to smaller groups on the wards. In addition, the staff gained practical experience and training in guided imagery and hypnosis as well as in naturopathic treatments over weekends and in half-day courses. This way, the staff learned about new treatment options and how and when to apply them. Knowledge was disseminated this way and new competencies were acquired (e.g., hypnosis for pain management, acupressure, etc.) and applied by nurses and physicians. Regular refresher teachings, including practical exercises to ensure quality of care, are being given.

In the process, it turned out to be essential to have one responsible, qualified physician, with additional qualifications in naturopathy and Traditional Chinese Medicine (TCM), who was able to anchor CAM deeper on the wards by making sure the new ways of treatment were being applied and by staff were being given repeated teachings. An outpatient clinic for CAM also was established. This is also according to the experience of other medical centers that emphasized on the advantage of having one protagonist leading the initiative in the hospital [19].

In order to publicize our institution’s new corporate strategy and promote its presence, different paths were taken, within and outside the hospital. Flyers and posters were designed and displayed in the building, and the hospital website now provides specific details of the CAM services offered and the new department now in operation. A consult service was established and in-house teachings were held regularly. Medical discharge reports now include CAM treatments applied on children, in a bid to inform primary care physicians and make a public statement about the new corporate policy. In addition, articles in magazines and journals were published and interviews videotaped (available online [24,25]). All these efforts help in communicating the significance and new philosophy of the hospital to its staff and the public.

Another important step was creating structures within the hospital. Wards were specialized in different areas like aromatherapy or homeopathy according to interests and competencies of the nurses assigned to the wards. For example, nurses are responsible for keeping a track of stocks of homeopathic remedies or aroma oils, teachings on other wards, etc. CAM was made part of every ward conference meeting held by these nurses. Furthermore, policies for specific treatments were established and were made readily available on the intranet for physicians and nurses to refer to and download. CAM consult services were established across the hospital, as well as a CAM clinic. A curriculum has also been compiled, which every staff member—new and old—is required to go through (it takes approximately 8 h).

## 5. Financing

The challenge of financing CAM services has been addressed as an issue in most institutions [19,21]. It is absolutely necessary to have enough philanthropic support or available funds at least in the early stages, as financial resources are often scarce and money is needed for salaries, education of staff, and to generate research projects. In our case, this turned out to be the biggest cost factor at the beginning, diminishing over time though, as most staff was trained and the new treatment methods became routine. Trained staff members can now partly assume the teaching of colleagues and therefore diminish the need of external, costly teachers. The clinical care component was self-supporting and not too expensive. In the US as well as in Germany, CAM services are mostly not covered by health insurances. Endowment funding therefore is essential to support sustainability. All of the biggest centers that offer CAM services in the US (e.g., Osher Centers at Harvard and Penny George Institute for Health and Healing) have several million dollars of endowment funding for support. In Germany, foundations can provide substantial monetary inputs. In addition, it turned out beneficial to negotiate special deals with insurance companies that at least many families now profit from. Cost effectiveness of integrative care over time has been demonstrated on numerous occasions [26,27,28].

## 6. Results of Our Project

Starting in 2015, complementary medicine was introduced step by step in the routine care of children and an integrative approach was established, which led to the foundation of an integrative medicine department with an attending physician in 2017. One of the first steps we took was identifying the resources present in the hospital, which we could draw upon. A questionnaire for the staff was designed and handed out to all staff members. Questions included the overall interest in the introduction of CAM treatments in the hospital as well as additional certifications of staff members. Seventy-seven percent of those who had answered the questionnaire reported having an interest in CAM methods. While most of them were nursing staff members (58%), 31% were physicians. Areas that garnered most interest were acupuncture, osteopathy, aromatherapy, and naturopathy. As these results will be published in a separate article, they will not be described in detail here.

In the feedback part of the questionnaire, interest in the research topic was evident through several remarks like:

- “I think it is wonderful that our hospital belongs to the more progressive ones. I support CAM treatments 100%.”
- “I am VERY happy, that CAM is now being established in our hospital and I hope that nursing staff can participate in trainings and teachings.”
- “I think this area is really interesting and important, I would love to receive additional training and participate.”

### CAM Services Now Offered at St. Marien

Apart from homeopathy, which has a long history at St. Marien, many other treatment modalities were integrated in the everyday care of the children at St. Marien, within three years of our project:

1. Aromatherapy is now being used regularly on hospitalized patients as roll-on sticks and in form of wraps and poultices. One nurse successfully completed her certification as an aromatherapist.
2. Acupuncture points have been taught to nurses and physicians and are now being applied in the form of pyonex press needles inserted in the acupuncture point P6 (pericard 6) to almost all children under anesthesia to prevent post-operative nausea and vomiting. Several studies have shown that P6 can be as effective as antiemetic drugs in preventing post-operative nausea and vomiting [29]. Nurses and doctors have been trained to insert the needles and regular refresher teachings take place.
3. Acupressure points are being taught to children and parents for cough, anxiety, abdominal pain, and nausea.
4. Another treatment modality is foot reflexology according to Hanne Marquardt [30]. Two indications were chosen—abdominal and lung conditions—and their treatment methods were taught to staff and parents. It is now being used regularly. A study was initiated and started in September 2016 to determine the effect of reflexology on children with these conditions.
5. Yoga is being offered in the hospital now and a study was initiated and completed by our research partner in Essen, Germany, about the effect of yoga for headaches in children.
6. Basic hypnotherapy methods (like “the magic glove”) were taught to physicians and nurses over the course of two weekends and are now being applied regularly.
7. Wraps and poultices are used in combination with aroma oils in the Newborn Intensive Care Unit (NICU) and the pediatric wards on patients with rheumatic diseases, anxiety, fever, joint pain, sleep problems, abdominal pain, and cough, among others.
8. Two doctors are now consulting on the wards on request for integrative treatment options for a variety of conditions. One of them is a certified homeopath, the other one is certified in naturopathy and TCM.
9. A “handling” course for parents has been held a few times since September 2016 to enable them to touch and move their babies in a more supportive and physiologic way to help their developmental process.
10. Herbal remedies are being applied and medical reference cards (detailing indications and dosage) have been designed for physicians.
11. A multidisciplinary pain management team now applies CAM Methods for treating pain in children.
12. Relaxation techniques of mind-body medicine are applied in the psychosomatic unit and social pediatric center.

## 7. Discussion

Integrating CAM methods into routine care for children within a hospital to create a real “integrative care” can be challenging. However, through our program, we demonstrated that CAM can successfully be integrated into a pediatric hospital in a relatively short time. The cornerstones that were essential to our process are summarized here and overlap some of which have been described as important components of other successful programs [18,19] as well as learned through personal communications (Kemper 2015).

1. The institution in which methods of the CAM spectrum require establishment has to make a clear decision on the path that should be taken.
2. Institutional support from the executive leader and medical director of the institution is essential. They represent the hospital and determine the overall concept of the institution. In our case, the leaders communicated well with each other about the new business concept, relayed this to their staff and put plans in place for it to be introduced by them. This enabled efficient evolution of the project and offered the prospect of sustainability.
3. It is important to have back-up support within the organization, as leaders can change over time. The project becomes vulnerable if the primary protagonist leaves. At least one well-respected person, preferably a qualified physician, should take the lead in the beginning. This person does not have to be an expert in all offered CAM modalities, but must be willing to cooperate with other CAM experts so that all team members can learn from each other. In St. Marien, two physicians led the project—one in the coordinating role and the other as an integrative physician present and active in the hospital.
4. It is crucial to have philanthropic support or available funds. Financial resources are often scarce and money is required for salaries, education of staff, and to generate research projects.
5. To minimize resistance, it is advisable to build on the prevalent interest of the institution. Depending on the organization, it could be research or clinical applications, or education, or a combination of both. It is easier to first follow that path and strengthen it before starting new ventures. In our case, clinical applications and education of staff were where the most interest was found, followed by research. We followed that interest, used existing resources, and tapped on motivation of the staff members (identified through a questionnaire) and subsequently built the new concept from inside out to keep staff members motivated.
6. It is essential to have motivated, creative colleagues working together, who can solve problems and find solutions one person can’t see.
7. Networking with like-minded colleagues and institutions creates motivation and inspiration. Sharing ideas and experiences can help expand the project. In our project, three hospitals were involved and common projects were created and ideas exchanged in regular meetings with all participants. Networking with international colleagues also created inspiration and offered new ideas.
8. It is important to not implement several modalities at once. To have a clear and simple treatment plan in which the patient can develop a relationship with the therapist is better that an overwhelming and expensive plan. In addition, too many new modalities can lead to an overload of the staff and therefore lead to resistance, which is not good for the project. We selected our modalities by identifying evidence-based knowledge and experience present within the hospital. Although there is a lack of evidence in the treatment of children, in accordance with other centers, we did not exclude modalities that had inadequate evidence [19]; instead, we evaluated efficacy by performing meta-analyses and reviews on various topics [31,32,33,34].
9. Multidisciplinary meetings can help broaden one’s spectrum of knowledge and experiences within the institution.
10. Research programs can be important in some institutions. Academic centers mostly want and need to add research to the project. Again, it is essential to follow the interest of the staff members and collaborate with other disciplines.
11. Education is an important aspect of any facility, be it in the form of lectures for students, training and rotations for staff members, or workshops.
12. Financing is a challenge in all centers. Financial plans have to be made, fundings organized, and payments by patients considered.
13. Cooperation and communication with colleagues in other hospitals as well as in private practices have to be established, in order to ensure complete integration into the field. In Germany, resistance can be quite high if the hospital offers treatments that colleagues in private practices don’t. This can hinder the success of the project. A qualified physician or well-respected person has to represent the project in order to raise acceptance and reputation of the institution.

Pediatric Integrative Medicine programs are found in several US medical centers and a few in Europe [18,19,35]. In 2009, nine US medical centers that played an important pioneer role in integrative medicine were interviewed on the critical factors leading to success or failure of the establishment of IM in hospitals in the fields of research, education, and clinical application [19]. Many of the factors discussed are consistent with our findings, and therefore can be considered essential in the process of implementing an integrative medicine program in any institution.

## 8. Conclusions

Integrative medicine can be established in a pediatric hospital, if there is support from leaders, has a motivated, multidisciplinary team with a qualified leader, and enough philanthropic or institutional initial funding. Regular team meetings to identify and pursue goals, communication of these within the institution, and repeated teaching modules that meet the interest of the staff are essential strategies to ensure motivation and sustainability. Also, the addition of CAM treatments into the curriculum of residents and nurses will allow for an even-deeper anchoring of integrative medicine into the institution. As a next step, new treatment standards and policies encompassing CAM treatments for various conditions can be defined, so they become a natural part of routine care of the hospital. Parental desire for CAM treatments is strong and a variety of indications are suitable for their application.

## Figures and Tables

**Table 1:** Existing staff expertise within St. Marien revealed through questionnaires.

Aromatherapy	Foot Reflexology	Massage
Art therapy	Homeopathy	Music therapy
Biofeedback	Naturopathy	Therapeutic riding
Craniosacral therapy	Nutritional counseling	Yoga
